# Replantation of a maxillary second molar after removal of a third molar with a dentigerous cyst: Case report and 12-month follow-up

**DOI:** 10.4317/jced.51291

**Published:** 2014-04-01

**Authors:** María A. Peñarrocha-Diago, Amparo Aloy-Prósper, David Peñarrocha-Oltra, Miguel Peñarrocha-Diago

**Affiliations:** 1 Associate lecturer in Oral Surgery. Medical and Dental School, University of Valencia, Valencia, Spain; 2 Master of oral surgery and implant dentistry. Medical and Dental School, University of Valencia, Spain; 3 Professor, Director of the Master’s Degree in Oral Surgery and Implant Dentistry. Medical and Dental School, University of Valencia, Spain

## Abstract

The aim of this study was to describe the replantation of a maxillary second right molar, which had been removed for surgical reasons in order to remove a dentigerous cyst associated with the adjacent third molar, and the case’s 12-month follow-up. A 51-year-old man presented swelling in the right maxillary area. Radiographic examination showed a large radiolucency in close proximity to the third molar, suggesting a follicular cyst. The third molar was extracted and the cyst underwent curettage. The second molar had to be extracted to enable complete removal of the cyst and to achieve primary closure of the wound, which would have been impossible without repositioning the molar. With this objective, extraoral endodontic treatment was performed, the root-end was resected and prepared with ultrasonic retrotips, and root-end filling was accomplished with MTA before the molar was replanted. At the 12-month follow-up, the tooth showed no clinical signs or symptoms, probing depth was no greater than 3 mm and radiographic examination showed no evidence of root resorption or periapical lesion.

** Key words:**Replantation, maxillary molar, follicular cyst, dentigerous cyst.

## Introduction

A dentigerous cyst can occasionally become extensive given that this type of lesion is usually asymptomatic; this situation makes treatment difficult because of associated teeth that may be impacted and placed at a considerable distance due to cyst pressure (1). The surgical approach required may endanger the vitality of adjacent teeth and even require their removal (2). In some cases, replantation of an extracted tooth may be possible, reinserting the tooth into its socket immediately following endodontic treatment and apical repair outside the oral cavity (3).

Cystic development is usually related to unerupted third molars or maxillary canines, and tends to displace the related tooth (2). A cyst formed around the maxillary third molar may invade the maxillary sinus, growing unnoticed to such an extensive size as to occupy a considerable portion of the maxillary sinus (1). As the cyst grows, the bony walls overlying the cyst thin out, giving rise to an egg shell sensation upon palpation and possibly transmitting pressure to the walls of the sinus, causing ophthalmologic and nasal symptoms to develop (4). The standard treatment for a dentigerous cyst involves surgical enucleation and extraction of the cyst-associated impacted or unerupted tooth. Sometimes the extension of the lesion may make require the removal of several adjacent teeth even though these are not directly related to the lesion (2). Replantation of some of these teeth may be considered, especially when the tooth removed has a strategic value, when periodontal conditions are favorable and when there is a possibility of restoration and adequate alveolar bone. Reviewing case reports published from January 1980 to September 2012, on intentional replantation of maxillary molars in humans, identified three cases (3,5,6). However, no article refers to the replantation of a tooth due to the extension of a dentigerous cystic involving an adjacent tooth, the subject of this case report.

This paper describes the replantation of a maxillary second right molar which was removed for surgical reasons in order to remove a dentigerous cyst associated with an adjacent third molar and the case’s 12-month follow-up.

## Case Reports

**Figure 1:**
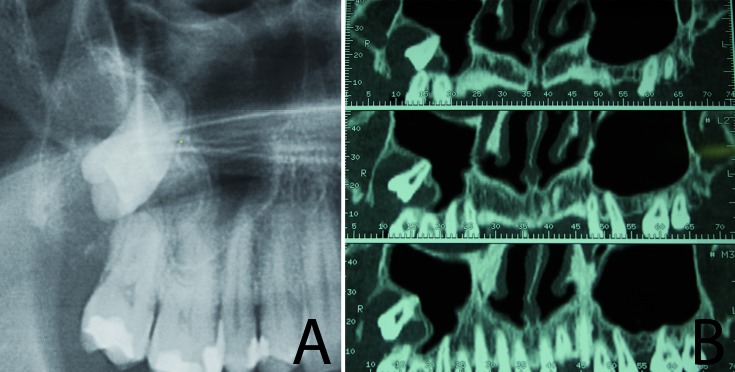
a.Panoramic radiograph showing a large radiolucency in close proximity to the third molar; b. Computed Tomography showing a radiolucent area around the third molar and comprising the second molar.

A 51-year-old man presented swelling and complained of discomfort and sensitivity in the maxillary right area. After his medical history had been examined, a panoramic radiograph of the area was taken, which showed a large well-defined radiolucent area in close proximity to the crown of the unerupted third molar extending to the second molar (Fig. 1). In the panoramic radiographic, the third molar was found in the right maxillary sinus, which suggested the presence of a follicular cyst. In order to determine the extent of the cyst, a computed tomography scan was performed (Fig. 1). This research was performed following the principles of the Declaration of Helsinki regarding research on humans. Written informed consent was obtained and the patient was scheduled for treatment.

**Figure 2:**
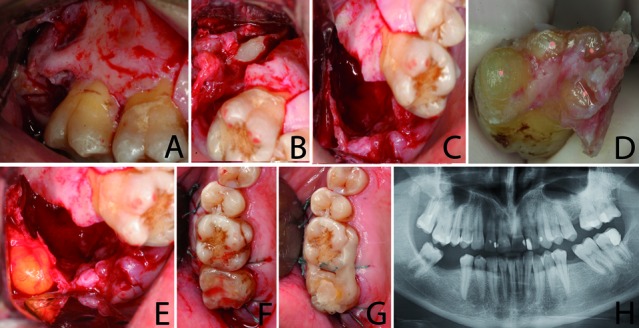
a.A full-thickness trapezoidal flap is raised; b.Extraction of the second and third molars; c.Cavity after curettage of the cystic lesion; d.Apical resection of the second molar’s roots; e.Picture showing the exposed buccal fat pad; f.Replacement of the second molar and suture; g.Splinting using composite resin; h.One-year radiographic follow-up.

Antisepsis was carried out with 0.12% chlorhexidine digluconate, and infiltrative anesthesia was administered with 4% articaine and adrenaline 1:100.000 (Inibsa®, Lliça de Vall, Barcelona, Spain). A mucoperiosteal flap was raised from mesial of the right first molar to distal of the second molar. Thinning and fenestration of the vestibular cortical bone were observed (Fig. 2). The third molar was extracted and the removal of the cyst commenced. However, the cyst reached around the roots of the second molar, which prevented complete curettage of the cavity (Fig. 2). The surgeon decided that the extraction of the second molar was necessary to ensure complete removal of the cyst (Fig. 2). The second molar was carefully extracted with forceps and no intraoperative complications occurred. The tooth was held in a sterile gauze sponge and the apices were beveled with a bur. With the tooth out of the socket, the root canals were biomechanically prepared and obturated with gutta-percha (Fig. 2). Root end cavities were prepared with ultrasonic retrotips, and filled with mineral trioxide aggregate (MTA) (ProRoot®, Dentsply, Tulsa, OK, USA). Before tooth replantation, the Bichat ball was extracted in order to place it inside the bone defect (Fig. 2). The tooth was replanted into its socket 30 minutes after its extraction; it was stable inside the alveolus. The flap was sutured with 3-0 silk sutures (Lorca-Marin®, Murcia, Spain) (Fig. 2). The tooth was left out of occlusion and splinted to the first molar with composite resin (Fig. 2). Amoxicillin (Clamoxyl®, GlaxoSmithKline, S.A, Madrid, Spain) 500 mg every eight hours for seven days, ibuprofen (Bexistar®, Laboratorio Bacino, Barcelona, Spain) 600 mg every eight hours for three days; and rinsing with 0.12% chlorhexidine digluconate (GUM®, John O. Butler Co, Chicago, USA) three times a day for seven days were prescribed during the postoperative period.

Histopathological study of the removed lesion identified it as a folicular cyst. The patient returned for a clinical follow-up two weeks following surgery (when the sutures and splint were removed), and after six weeks. A panoramic radiograph was taken after twelve months: the root surface and periodontal ligament appeared intact. The postoperative period was uneventful and the replanted tooth was asymptomatic.

At the 12-month follow-up the tooth showed no clinical signs or symptoms and probing depth was no greater than 3 mm. No evidence of root resorption or periapical lesion was observed radiographically (Fig. 2).

## Discussion

This study describes the replantation of a maxillary second right molar, which had been removed for surgical reasons in order to achieve a complete removal of a dentigerous cyst associated with an adjacent third molar. Dentigerous cysts are the most common type of developmental odontogenic cyst; these cysts are the second most common cystic lesions of the jaws, after radicular cysts (7). As they are usually asymptomatic, a cyst can become extensive and involve adjacent teeth. Surgical approach is not always easy, since associated teeth are often impacted and placed at considerable distance due to pressure from the cyst (1). The standard treatment for a dentigerous cyst is enucleation and extraction of the cyst-associated impacted or unerupted tooth (8). A Caldwell-Luc cannot be performed as a surgical window cannot be made distal of the second molar roots, and making a more anteriorly positioned window would compromise the access and visibility needed to perform cystectomy and third molar extraction. Furthermore, in this case, the third molar was below the floor of the maxillary sinus and the cyst did not affect the maxillary sinus, as can be seen in the CT scan. As the second molar buccal cortical bone was intact, replantation of the second molar was planned following endodontic treatment and apical repair outside the oral cavity.

Splinting is necessary following replantation in order to reduce mobility of the tooth and aid the initial periodontal healing (9). According to several authors, replanted teeth should be splinted only for a short period (one to two weeks) since a longer time could result in ankylosis or root resorption (9). Some authors have used semi-rigid splinting with wire or silk (5,10), while other authors prefer rigid splinting using, for example, composite resin (11). In this case, a composite rigid splint was used and removed two weeks after replantation.

The most frequent complications of replantation are root resorption and ankylosis (12,13). Ankylosis is directly related to the amount of time the tooth is out of the mouth during the procedure. Most resorptive processes are diagnosed within the first two-to-three years, although they can occur even after five or ten years (15). Raghoebar and Vissink (6) replanted 29 molars, and reported a rate of ankylosis of 72% (4 cases) after 11 years. In the present case, there was no evidence of ankylosis at the 12-month follow-up, the replanted tooth showed a normal-appearing periodontal ligament and the patient did not report any pain.

The Bichat’s ball can be used to treat large jawbone defects (14,15). Alkan *et al*. (13) and Martin-Granizo *et al*. (14) used the buccal fat pad in 26 and 29 patients respectively, to treat defects resulting from tumor excisions, maxillary cysts and oro-antral communications; the epithelization process started during the first week and was complete after 30 or 40 days; none of the patients suffered aesthetic disturbances, limited mouth opening or facial paralysis. Zhong *et al*. (15) utilized the Bichat’s ball to reconstruct maxillary defects after partial maxillary resection in 38 patients; three cases developed fistulas in the oral cavity. After 1-12 years follow-up, there was normal mouth opening (not less than 37 mm), facial contour symmetry and patients were rehabilitated with removable partial dentures. In this study, the Bichat’s ball was extracted before the tooth replantation and placed inside the bone defect.

The success of replantation, estimated as the tooth retention rate, has been reported on average to be between 67% and 93.7% (6,13). In this case, the second molar was replanted successfully with no signs of failure after the twelve-month follow-up.

## Conclusions

In the surgical approach to remove an extensive cystic lesion, some teeth can be sacrificed; replantation may be considered in order to maintain a tooth’s original position, especially when it has a strategic value, when periodontal conditions are favorable and when there is a possibility of restoration and adequate alveolar bone.
